# Risk factors predicting need for the pediatric intensive care unit (PICU) post-hematopoietic cell transplant, PICU utilization, and outcomes following HCT: a single center retrospective analysis

**DOI:** 10.3389/fped.2024.1385153

**Published:** 2024-04-16

**Authors:** Amanda K. Johnson, Sinziana Cornea, Samuel Goldfarb, Qing Cao, Julia A. Heneghan, Ashish O. Gupta

**Affiliations:** ^1^ Department of Pediatrics, Division of Blood and Marrow Transplantation & Cellular Therapy, University of Minnesota MHealth Fairview Masonic Children’s Hospital, Minneapolis, MN, United States; ^2^ Department of Pediatrics, Division of Pulmonology, University of Minnesota MHealth Fairview Masonic Children’s Hospital, Minneapolis, MN, United States; ^3^ Biostatistics Core, University of Minnesota Masonic Cancer Center, Minneapolis, MN, United States; ^4^ Department of Pediatrics, Division of Pediatric Critical Care Medicine, University of Minnesota MHealth Fairview Masonic Children’s Hospital, Minneapolis, MN, United States

**Keywords:** hematopoietic cell transplant (HCT), hematopoietic stem cell transplant (HSCT), pediatric intensive care unit (PICU), utilization, outcomes, pulmonary function tests (PFTs)

## Abstract

Hematopoietic cell transplant (HCT) is a curative treatment for multiple malignant and non-malignant disorders. While morbidity and mortality have decreased significantly over the years, some patients still require management in the pediatric intensive care unit (PICU) during their HCT course for additional respiratory, cardiovascular, and/or renal support. We retrospectively reviewed pediatric patients (0–18 years) who underwent HCT from January 2015–December 2020 at our institution to determine risk factors for PICU care and evaluate PICU utilization and outcomes. We also assessed pulmonary function testing (PFT) data to determine if differences were noted between PICU and non-PICU patients as well as potential evolution of pulmonary dysfunction over time. Risk factors of needing PICU care were lower age, lower weight, having an underlying inborn error of metabolism, and receiving busulfan-based conditioning. Nearly half of PICU encounters involved use of each of respiratory support types including high-flow nasal cannula, non-invasive positive pressure ventilation, and mechanical ventilation. Approximately one-fifth of PICU encounters involved renal replacement therapy. Pulmonary function test results largely did not differ between PICU and non-PICU patients at any timepoint aside from individuals who required PICU care having lower DLCO scores at one-year post-HCT. Future directions include consideration of combining our data with other centers for a multi-center retrospective analysis with the goal of gathering and reporting additional multi-center data to work toward continuing to decrease morbidity and mortality for patients undergoing HCT.

## Introduction

Hematopoietic cell transplant (HCT) is a potentially curative therapy for a variety of conditions including hematologic malignancies, non-malignant hematologic disorders, immunodeficiencies, and several inherited metabolic disorders. Over the past several decades, there have been significant advances resulting in lower rates of relapse, graft failure, and other complications with resultant improved survival (1, 2). Despite this, there is still morbidity and mortality from transplant-related complications in the peri-transplant period. Pediatric intensive care unit (PICU) utilization during HCT in general is known to increase the risk of mortality post-HCT, particularly if it involves multisystem organ failure (3). However, it is unclear which specific components of PICU care (e.g., any respiratory support vs. intubation, types of dialysis) in more recent years are associated with the greatest risk for different short- and long-term outcomes (3–5). Data from a variety of prior studies suggests that stem cell transplant patients and immunocompromised patients have higher mortality than other critically ill children post-intubation and have higher mortality when they are difficult to oxygenate, require high frequency oscillatory ventilation and/or are fluid overloaded (6–9). Further risk assessment remains challenging given advances in practice and supportive care changing rapidly over time in addition to institutional variability in HCT and PICU practices.

To date, some single-center retrospective studies of risk factors for PICU care post-HCT have been published (3–5) and multiple older prospective and multi-center studies also available (10–12). However, recent prospective and multi-center experiences are lacking aside from two recently published multi-center retrospective studies (13, 14). Further, utilization of different supports in the PICU (e.g., types of respiratory support, types of dialysis) during HCT needs to be better described as HCT centers often have different approaches and institutional practices for interventions necessitating a PICU transfer and ongoing PICU care. Additionally, information linking long-term outcomes to physiologic instability during the HCT process is not readily available. For pulmonary dysfunction in particular, delay in diagnosis of later complications and dysfunction post-HCT, specifically chronic graft vs. host disease involving the lungs, has led to creation of the TRANSPIRE study (NCT04098445) which aims to enhance and develop further screening and early detection of late pulmonary complications post-HCT to improve the post-HCT management (15). Herein, we report our single center experience of transplant related risk factors for PICU care and utilization as well as ascertain short- and long-term outcomes after PICU care. Additionally, we aim to further characterize long-term lung disease by comparing pulmonary function test (PFT) results of individuals throughout their HCT course.

## Methods

We retrospectively reviewed pediatric patients (0–18 years) who underwent HCT at the University of Minnesota from January 2015–December 2020. HCT and PICU databases were queried for demographic data in addition to pre-HCT, HCT and post-HCT data. These databases are prospectively collected and longitudinally monitored with data validity and quality checks. For the PICU data, local data was obtained from the Virtual Pediatric Systems (VPS, LLC). VPS is a prospectively collected cohort of consecutive PICU admissions and chart abstraction is undertaken by a trained coordinator. Variables evaluated included type of respiratory support and duration as well as need for dialysis. Only PICU encounters which occurred after the transplant date were assessed. Further information was extracted from the electronic medical record via chart review for missing data.

For PFT data, number of PFTs per patient and timing of PFTs in relation to HCT were evaluated (baseline prior to HCT, 100 days post-HCT, 6 months post-HCT, 1-year post-HCT, and greater than 1-year post-HCT). PFTs were assigned to the category closest to their time of completion (80–110 days for 100 days post-HCT, 130–281 days for 6 months post-HCT, and 328–499 days for 1-year post-HCT). Variables assessed on PFTs included forced vital capacity (FVC), forced expiratory volume in one second (FEV1), FEV1/FVC, mid-forced expiratory flow volumes between 25% and 75% of vital capacity (FEF25–75), forced residual capacity (FRC), residual volume (RV), total lung capacity (TLC), and diffusing capacity for carbon dioxide (DLCO). PICU and non-PICU patient PFT data were then imported into the Global Lung Function Initiative (GLI) Calculator (https://gli-calculator.ersnet.org/) to determine z-scores and assess for statistically significant differences with alpha level of 0.05.

### Statistical methods

University of Minnesota’s pediatric transplant and PICU databases were queried for pediatric allogeneic HCTs and PICU encounters from January 2015–December 2020. Descriptive statistics, including mean, median, IQR, and range, were employed to characterize demographic, clinical, laboratory variables and the Global Lung Function Initiative (GLI) calculated PFT data across the two groups. Categorical variable comparisons between groups were conducted using chi-square test or Fisher’s exact test in cases of limited expected counts, and continuous variable comparisons between groups were conducted using Wilcoxon rank-sum tests. Overall survival was estimated by Kaplan-Meier curves (16). Log-rank test was used to compare the estimates between groups. Multivariate logistic regression analysis was also conducted to further characterize risk factors for PICU admission post-HCT. Variables with *p* < 0.1 in univariate analysis were evaluated for multivariate analysis (Supplementary Tables S1, S2). Odds ratios were determined with odds ratios >1 indicating more risk of needing PICU care.

All statistical tests were two-sided, and significance was established at *p* < 0.05. The statistical analyses were conducted using SAS 9.4 (SAS Institute, Inc., Cary, NC) and R version 4.2.2 (R Foundation for Statistical Computing, Vienna, Austria).

## Results

A total of 277 children underwent HCT between January 2015 and December 2020. Median age at time of transplant was 6.2 years (IQR 1.1–11.4 years). Over half of children were male (58.1%) with most children of White race (76.5%). Most common indications for HCT were non-malignant disorders (41.2%), followed by malignant disorders, and underlying inborn errors of metabolism (Table 1). Umbilical cord blood was the predominant donor source (42.2%), followed by matched unrelated donor, and matched sibling donor (Table 1).

**Table 1:** Demographic and clinical characteristics of included patients.

Characteristics	PICU	No PICU	*P* value
*N*	77	200	
Number of PICU Encounters			–
1	51 (66.2%)	–	
2	14 (18%)	–	
3	12 (16%)	–	
Day of Transplant to 1st PICU Event (Days)			–
Median (IQR)	51 (310)	–	
Age at Transplant (years)			
Median (IQR)	2.5 (8.8)	7.1 (10.4)	<0.01
Weight (kg)			
Median (IQR)	13.3 (20.9)	22.5 (31.4)	<0.01
BMI (kg/m^2^)			
N	76^a^	200	0.25
Median (IQR)	17.2 (3.6)	17.6 (4.6)	
BMI Group			
≤25	72 (94.7%)	182 (91.0%)	0.31
>25	4 (5.3%)	18 (9.0%)	
Race			0.02
Unknown	5 (6.5%)	23 (11.5%)	
Caucasian	58 (75.3%)	154 (77.0%)	
African American	3 (3.9%)	15 (7.5%)	
American Indian	2 (2.6%)	3 (1.5%)	
Asian	9 (11.7%)	5 (2.5%)	
Gender			0.84
Male	44 (57.1%)	117 (58.5%)	
Female	33 (42.9%)	83 (41.5%)	
Underlying Disease			0.01
Inherited metabolic disorders	29 (37.7%)	43 (21.5%)	
Non-malignant	23 (29.9%)	91 (45.5%)	
Malignant	25 (32.5%)	66 (33.0%)	
Cell source			0.20
Bone marrow	35 (45.5%)	110 (55.0%)	
PBSC	3 (3.9%)	12 (6.0%)	
UCB	39 (50.7%)	78 (39.0%)	
Donor Type (Bone marrow + PBSC)			0.18
Matched sibling	10 (26.3%)	49 (40.2%)	
Matched URD	19 (50.0%)	42 (34.4%)	
Mismatched Sibling/URD + Haploidentical	9 (23.7%)	31 (25.4%)	
CMV matching			0.04
Donor+ Recipient+	5 (6.5%)	38 (19.0%)	
Donor+ Recipient-	8 (10.4%)	11 (5.5%)	
Donor- Recipient+	36 (46.8%)	79 (39.5%)	
Donor- Recipient-	28 (36.8%)	72 (36.0%)	
Conditioning Regimen			0.01
Cyclophosphamide + Fludarabine + TBI	9 (11.7%)	50 (25.2%)	
Cyclophosphamide + TBI	14 (18.2%)	39 (19.7%)	
No TBI	4 (5.2%)	23 (11.6%)	
Busulfan with no TBI	50 (64.9%)	86 (43.4%)	
LPS/KPS			0.20
≤80	23 (29.9%)	45 (22.5%)	
>80	54 (70.1)	155 (77.5%)	
HCT-CI			0.32
0	39 (50.7%)	121 (60.5%)	
1–2	24 (31.7%)	48 (24.0%)	
>=3	14 (18.2%)	31 (15.5%)	
DRI			0.54
Very Low	0	5 (2.5%)	
Low	12 (15.6%)	33 (16.5%)	
Intermediate	12 (15.6%)	21 (10.5%)	
High	1 (1.3%)	2 (1.0%)	
Very high	52 (67.5%)	134 (67.0%)	
Missing	0	5 (2.5%)	
Cardiopulmonary comorbidity prior to HCT			0.74
Yes	14 (18.2%)	33 (16.5%)	
No	63 (81.8%)	167 (83.5%)	
CMV status			0.86
Yes	20 (26.0%)	54 (27.0%)	
No	57 (74.0%)	146 (73.0%)	
EBV status			0.27
Yes	11 (14.3%)	40 (20.0%)	
No	66 (85.7%)	160 (80.0%)	
Adenovirus status			<0.01
Yes	10 (13.0%)	7 (3.5%)	
No	67 (87.0%)	193 (96.5%)	
Adenovirus time (days)			
N	10	7	
Median (range)	77 (71)	76 (62)	0.47
Transplant year			0.33
2015	12 (15.6%)	41 (20.5%)	
2016	20 (26.0%)	32 (16.0%)	
2017	13 (16.9%)	37 (18.5%)	
2018	11 (14.3%)	33 (16.5%)	
2019	14 (18.2%)	28 (14.0%)	
2020	7 (9.1)	29 (14.5%)	
ANC engraftment status at last follow-up			0.50
Alive without engrafted	0	1 (0.5%)	
Dead without engrafted	2 (2.6%)	2 (1.0%)	
ANC > 500 × 3 days	75 (97.4%)	197 (98.5%)	
Follow-up Status			<0.01
Alive	40 (52.0%)	181 (90.5%)	
Dead	37 (48.0%)	19 (9.5%)	
Alive patients follow up time (days)			<0.01
N	40	181	
Median (IQR)	1,790.5 (737.5)	1,812 (1,067)	
Relapse status at last follow-up			<0.01
Alive without relapse	37 (48.1%)	173 (86.5%)	
Dead without relapse	32 (41.6%)	7 (3.5%)	
Relapse	8 (10.4%)	20 (10.0%)	

BMI, body mass index; UCB, umbilical cord blood; LPS, Lansky Performance Score; KPS, Karnofsky Performance Score; HCT-CI, hematopoietic cell transplant comorbidity index; DRI, disease-risk index; CMV, cytomegalovirus; EBV, Epstein-Barr virus; ANC, absolute neutrophil count.

^a^ One patient had missing height information and BMI unable to be calculated.

### PICU admission and resource use

A total of 77 children (38.5%) needed PICU care. Of these, most patients had one PICU stay (66.2%). Median time from HCT to first PICU admission was 51 days (IQR 13–323 days).

### Univariate analysis

Patients needing PICU care were more likely to be younger (*p* < 0.01). Lower weight was also associated with higher risk of PICU admission (*p* < 0.01). BMI was not significantly different between the two groups. Patients with underlying inborn errors of metabolism were more likely than individuals with non-malignant or malignant underlying diagnoses to need PICU care (*p* = 0.01). Patients with busulfan-based conditioning regimens were also more likely to need PICU care (*p* = 0.01). Individuals with underlying pulmonary and/or cardiac disease (moderate or severe pulmonary co-morbidity sub score, arrhythmia, heart valve, other cardiac condition) were not found to be at a higher risk to need PICU care. Donor type was not a significant risk factor for PICU care. Potential metrics of patient complexity or fragility (i.e., performance status, comorbidity index [HCT-CI], and disease-risk index [DRI]) were not associated with needing PICU care.

Patients that required PICU care were more likely to have detectable adenoviremia at some point during their HCT course (*p* < 0.01), while there was no difference in detectable CMV and EBV viremia between the two groups. CMV matching was different amongst the two groups with donor negative, recipient positive status noted in the highest proportion of individuals who needed PICU care. Additional demographic and clinical data can be found in Table 1.

### Multivariate analysis

Based on univariate analysis results (Table 1), age, weight, race, underlying diagnosis, CMV matching and conditioning regimen were considered for multivariate analysis (Supplementary Tables S1, S2). A final multivariate model incorporating both age and underlying diagnosis found that for every one year increase in age while controlling for underlying diagnosis, the odds of needing PICU care decreased (odds ratio 0.92, 95% CI 0.87–0.97).

Of the 77 individual patients who received PICU care, there were a total of 127 ICU encounters. Median length of PICU stay was 5.1 days (IQR 1.6–15.9 days). Most patients were neutrophil engrafted prior to PICU stay (83.5%), while some achieved neutrophil engraftment following PICU admission (15.0%). A small proportion of these patients failed to engraft (1.6%). Additional details of PICU encounters are listed in Table 2.

**Table 2:** Characteristics of PICU encounters.

Factor	PICU Encounters (*N* = 127)
Time from BMT to PICU (days)	
Median (IQR)	87 (292)
Engraftment status at PICU admission	
Engrafted before PICU	106 (83.5%)
Engrafted after PICU	19 (15.0%)
Failed to engraft	2 (1.6%)
Age at time of PICU Encounter (Years)	
Median (IQR)	3.9 (9.3)
Weight at time of PICU Encounter (kg)	
Median (IQR)	15.7 (19.8)
PICU_PRISM_3	
Median (IQR)	10.0 (10.0)
PIM_3 Logit	
Median (IQR)	−2.6 (1.5)
Tracheostomy	
No	126 (99.2%)
Yes^a^	1 (0.8%)
HFNC	
Yes	59 (46.5%)
No	68 (53.5%)
HFNC Duration (Days)	
N	59
Median (IQR)	1.1 (1.4)
ECMO	
No	124 (97.6%)
Yes	3 (2.4%)
ECMO Duration (Days)	
N	3
Median (IQR)	10.8 (20.6)
NIPPV	
Yes	66 (52.0%)
No	61 (48.0%)
NIPPV Duration (Days)	
N	66
Median (IQR)	3.4 (5.2)
MV	
Yes	65 (48.8%)
No	62 (51.2%)
MV Duration (Days)	
N	65
Median (IQR)	8.9 (12.4)
RRT	
Yes	22 (17.3%)
No	105 (82.7%)
RRT Duration (Days)	
N	22
Median (IQR)	10.5 (21.3)
PICU LOS (Days)	
Median (IQR)	5.1 (14.3)

Organ support duration above only includes that while in PICU.

HFNC, high flow nasal cannula; ECMO, extracorporeal membrane oxygenation; NIPPV, non-invasive positive pressure ventilation; MV, mechanical ventilation; RRT, renal replacement therapy; LOS, length of stay.

^a^ Additional details of this patient are not included as they may be identifying.

Regarding respiratory support utilization, multiple modalities were utilized in the PICU with some patients utilizing more than one modality. In approximately half of encounters (46.5%), high flow nasal cannula support (HFNC, which can commonly occur in the general ward in our hospital) was used, non-invasive positive pressure support (NIPPV) was used in over half of encounters (52.0%), and mechanical ventilation (MV) was utilized in just under half of encounters (48.8%). Median duration of HFNC support was 1.1 days (IQR 0.5–1.9 days) while median duration of NIPPV was 3.4 days (0.8–6 days), and median duration of mechanical ventilation was 8.9 days (IQR 2.9–15.3 days). Additional organ support included extra-corporeal membrane oxygenation (ECMO) in three encounters (2.4%), with a median duration of 10.8 days (IQR 3.5–24.1 days) and renal replacement therapy in 22 encounters (17.3%), with a median duration of 10.5 days (IQR 0.6–21.9 days).

### Mortality outcomes

**Figure 1:**
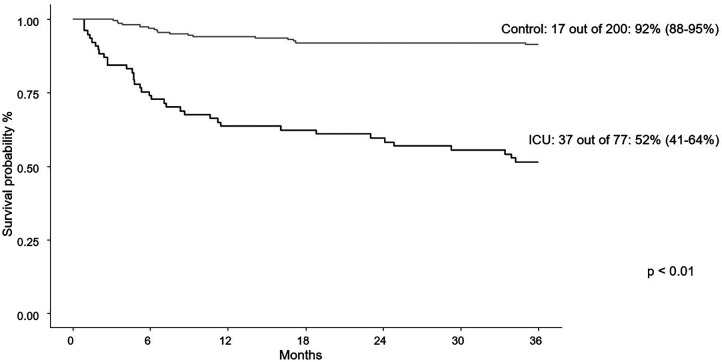
Three-year survival curves for first PICU encounter vs. control patients.

**Figure 2:**
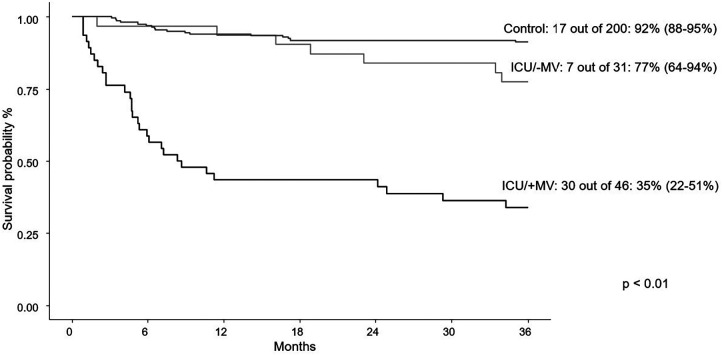
Three-year survival curves for first PICU encounter +/− mechanical ventilation (MV) vs. control patients.

**Figure 3:**
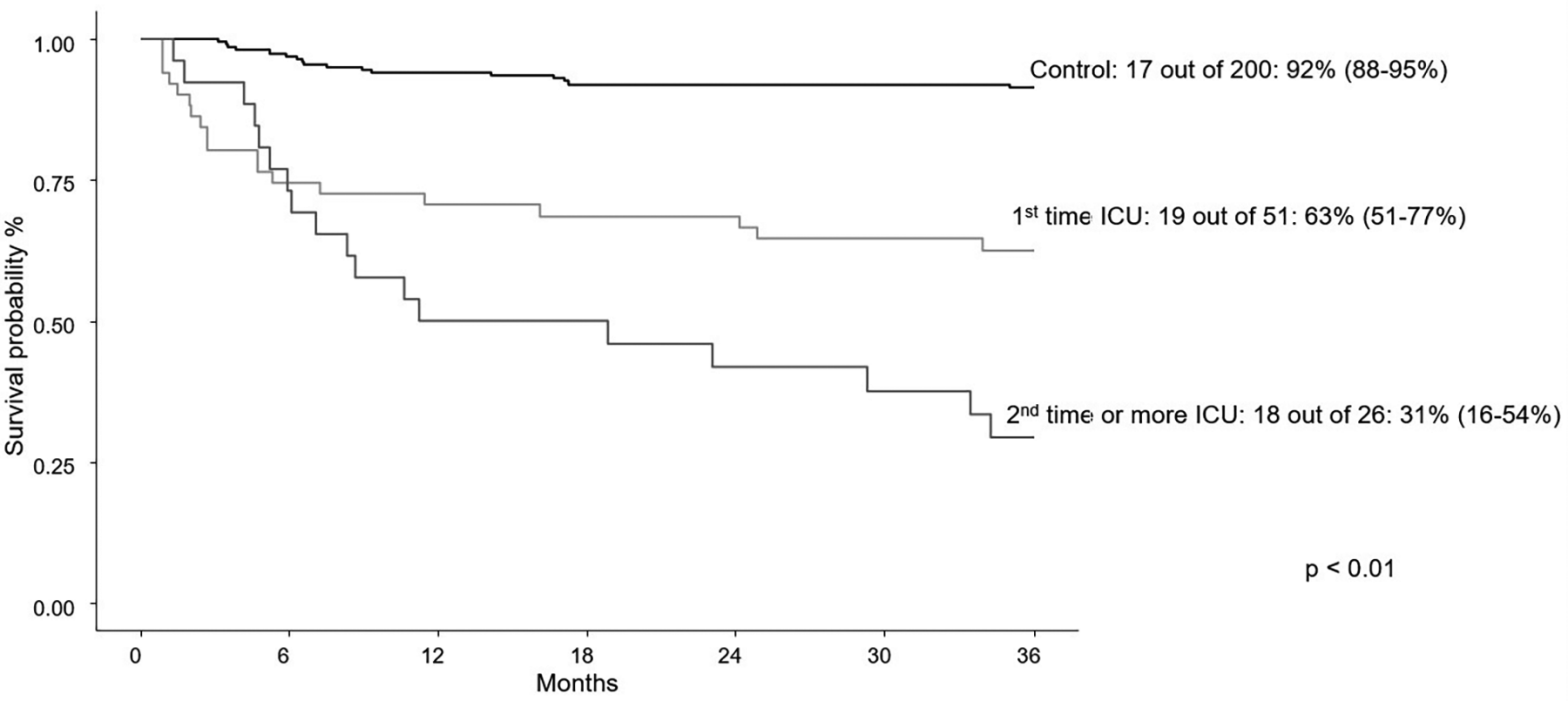
Three-year survival curves for PICU encounter(s) vs. control.

At last follow-up, individuals needing PICU care experienced higher mortality, but had similar rates of relapse when compared to patients not needing PICU care (Table 1). Overall survival at three years was 51% in PICU group and 92% in non-PICU group (*p* < 0.01; Figure 1). When PICU care was combined with the need for mechanical ventilation, a further decrease in overall survival at three years was found (77% without mechanical ventilation and 35% with mechanical ventilation; Figure 2). More than one PICU stay was also found to negatively impact three-year overall survival (63% for single PICU stay and 31% for two or more PICU stays; Figure 3). Lastly, all three patients where ECMO was utilized died.

### Pulmonary function tests (PFTs)

Of the total cohort of 277 patients, 108 patients completed PFTs at any time point. The 169 patients that were excluded from PFT analysis did not have PFTs at any time point pre-BMT and up to one-year post-BMT. Of these 169 patients, 131 were less than 6 years-old at time of BMT and unable to complete PFTs due to age. Pulmonary function testing was completed for 91 patients at baseline, 21 patients at Day 100 post-HCT, 9 at six months post-HCT, 74 at one-year post-HCT, and 59 patients had PFTs after one-year post-HCT. Of note, 6 patients of the 108 patients who completed any PFTs died prior to the six-month post-HCT timepoint.

There were no statistically significant differences in the PFT variables of interest between PICU and non-PICU patients at baseline (Supplementary Table S3). At one-year post-HCT, DLCO was lower in patients who required PICU support during HCT (*p* = 0.03; Supplementary Table S4). At all other timepoints, there were no statistically significant differences in PFT variables between PICU and non-PICU patients.

## Discussion

In this single-center retrospective review of nearly 300 pediatric patients undergoing HCT during the study period, we demonstrated that a variety of risk factors are associated with need for PICU care post-HCT as well as demonstrating the importance of younger age increasing risk for need for PICU care when controlling for underlying disease. We also highlighted that NIPPV and mechanical ventilation were the most utilized PICU therapies by HCT patients transferred to the PICU post-HCT as well as reviewed outcomes of post-HCT patients who needed PICU care. Finally, we demonstrated a difference in diffusing capacity measurements at one-year post-HCT in patients admitted to the PICU. Spirometry and plethysmography findings did not demonstrate any statistically significant changes from pre-transplant studies to post-transplant follow-up. Our study and its results add to the growing body of literature to inform the care and management of HCT patients going forward.

Our study found that younger patients (and patients with lower weights) were more likely to need ICU care, which is not surprising given patients with smaller airways have a higher risk of increased respiratory needs and intubation post-HCT (17). Our cohort had a higher proportion of patients needing PICU care compared to previously reported retrospective cohorts (38.5% vs. 10%–15%; 3–5, 13, 14) and is likely the result of our patient cohort being younger, lower weight and having a high proportion of patients with underlying inborn errors of metabolism, who are also have a higher risk of complications peri-transplant (17–19). Further, when evaluating PICU care risk by multivariate logistic regression and adjusting for underlying disease, increasing age was found to have a lower odds of needing PICU care, which is in keeping with lower age (and likely smaller weight patients) experiencing higher risk of PICU need peri-HCT (17).

Additionally, by univariate analysis, racial identity differences were noted with a higher portion of Asian individuals utilizing PICU care. While the racial demographics in our study are felt to closely approximate our geographic area, the small numbers of non-White individuals and our study occurring at a single center make this data difficult to interpret. Further, race is likely a proxy for a variety of other factors and putting significant weight on race, which is largely now accepted as a social construct, is not appropriate. However, race could be a more important factor in multi-institutional studies with more granular multivariate analyses that consider socioeconomic status, proximity to transplant center, insurance status, etc.

A variety of respiratory supports were the most utilized PICU resources in our study with escalating durations of support with more invasive modalities of respiratory assistance. The relatively short PICU stay duration of 5.1 days is likely influenced by high percentage of patients transferring to PICU for HFNC and NIPVV support. The median time to first PICU admission was 51 days post-HCT in our cohort. While that timing post-HCT explains the high percentage of patients who were neutrophil engrafted prior to PICU transfer, it also illustrates that significant organ dysfunction can occur weeks following engraftment, including after discharge from the initial HCT hospitalization. This is consistent with reports from a recent multi-center study, which demonstrated ICU exposure for patients post-HCT increased across the measured time points of day +100, 1-year and 5-years post-HCT (14). Additionally, our institutional practice of allowing advanced respiratory support on the HCT unit (HFNC and some NIPPV) potentially led to PICU transfer later in the post-HCT course than in institutions with more restrictive policies. Post-HCT PICU 3-year overall survival from our cohort (52.0%) is higher in comparison to a recently published study (14.9%; 13) that evaluated post-HCT outcomes and is similar to another recent multi-center study’s 1-year survival post-ICU transfer (52.5%; 14). This discrepancy is at least partially due to improvement in supportive care over time. However, it should also be noted that despite our higher proportion of patients needing PICU care compared to other cohorts, survival in our cohort is at least the same or better than in other studies, highlighting our PICU’s management of our patients and our comfort as a transplant center taking higher risk HCT patients.

Our study demonstrated no significant differences between longitudinal pulmonary function testing in children exposed to the PICU during their transplant course compared to those who were not aside from lower DLCO at one-year post-HCT in children exposed to PICU (Supplementary Tables S3, S4). It is also important to specifically note around one-third of patients had an abnormal DLCO at baseline, but DLCO abnormality increased at one-year post-HCT for patients who required PICU care during the peri-HCT period (Supplementary Tables S3, S4). Decreased diffusing capacity demonstrates a decline in gas exchange with parenchymal lung changes consistent with pulmonary interstitial lung disease. In our cohort, we did not demonstrate an association between obstructive or restrictive lung disease after PICU admissions. The PFT data are limited in number and highlights the importance of studies like TRANSPIRE to further evaluate standard PFTs at multiple time points as well as evaluate additional markers of lung dysfunction in the HCT setting (15). Due to the need for patients to be developmentally capable of participating in PFTs, which generally are difficult to perform in children under 6 years of age, alternative measurements are being explored. Some of these include airway oscillometry (which is performed during normal tidal volume breathing, and therefore does not require the same level of participation) and multiple breath washout to determine lung clearance index (a testing modality that requires only tidal breathing), which are being evaluated in TRANSPIRE and are important for future study in this cohort (15, 20, 21). Despite our limited PFT data, there is a paucity of this data published in the HCT population, and having serial PFTs for some HCT patients is a strength of our study.

Given the retrospective nature of our study, there are study limitations. As one example of retrospective limitations in our study, adenoviremia was found to be associated with higher risk for PICU care. However, we do not have data on additional details of adenoviremia (sites of involvement, highest viral load, end-organ dysfunction thought to be specifically related to adenoviremia, etc.). Additionally. transplant-related risk factors and PICU utilization and outcomes can have inherent institutional variability requiring larger and multi-center studies to establish stronger associations. There is also an inherent limitation of lack of availability of PFT data in the younger cohort who are typically not developmentally capable of performing standard PFTs until they reach 6 years of age, so other modalities should continue to be explored and used to address this concern.

Understanding the risk factors, peri-HCT PICU utilization and long-term pulmonary outcomes in children undergoing HCT is critical to develop a long-term monitoring and management plan. As transplant conditioning and post-transplant management continues to evolve, a deeper understanding and assessment is critical for optimal pre- and post-transplant care. Along with other recent studies (3–5, 13, 14), creation of a pre-HCT PICU and outcome risk scoring system as more studies are published would be an important tool for clinicians to predict outcomes for pediatric HCT patients.

## Data Availability

The raw data supporting the conclusions of this article will be made available by the authors, without undue reservation.
