# Supplementary material for: Risk factors predicting need for the pediatric intensive care unit (PICU) post-hematopoietic cell transplant, PICU utilization, and outcomes following HCT: a single center retrospective analysis
Source: Front Pediatr. 2024 Apr 16;12:1385153. doi: 10.3389/fped.2024.1385153 (PMC11059064; doi:10.3389/fped.2024.1385153)
Supplement: Supplementary file 1 [file Table1.pdf]

	**Age**	**Weight**	**Race**	**Underlying dx**	**CMV**	**Regimen**
**Age**	--	--	--	--	--	--
**Weight**	<0.01	--	--	--	--	--
**Race**	0.76	0.30	--	--	--	--
**Underlying dx**	0.09	0.30	0.14	--	--	--
**CMV**	0.04	0.01	<0.01	<0.01	--	--
**Regimen**	<0.01	<0.01	0.33	<0.01	<0.01	--

Supplementary Table 1. Correlation Table for Multivariate Analysis.
Dx = diagnosis, CMV = cytomegalovirus, Regimen = Conditioning Regimen

	**Odds ratio (PICU) 95% CI**	**P value**
**Age (1 year older)**	0.92 (0.87-0.97)	**<0.01**
**Underlying Disease**		0.09
Malignant	1.00	
Non-malignant	0.55 (0.28-1.08)	
Inherited Metabolic Disorders	1.10 (0.53-2.29)	

Supplementary Table 2. Final Multivariate Analysis Model.
CI = confidence interval
Interpretation: Every one year older was significantly associated (p < 0.01) with odds ratio of 0.92 (95% CI 0.87-0.97), less chance of experiencing PICU after adjusted with underlying disease. Underlying disease was associated with marginal association with probability of having PICU (p = 0.09).

	**All Patients**	**No ICU**	**ICU**	**P-value**
	N=91	N=76	N=15	
**DLCO_Z**				0.69
Normal: |z| <1.64	54(59.3%)	45(59.2%)	9(60.0%)	
Mild: |z| [1.64-3)	31(34.1%)	25(32.9%)	6(40.0%)	
Moderate:|z| [3,4)	3(3.3%)	3(3.9%)	0	
Missing	3(3.3%)	3(3.9%)	0	
**TLC_Z**				0.95
Normal: |z| <1.64	75(82.4%)	62(81.6%)	13(86.7%)	
Mild: |z| [1.64, 3)	12(13.2%)	10(13.2%)	2(13.3%)	

Missing	4(4.4%)	4(5.3%)	0	
**RV_Z**				0.29
Normal: |z| <1.64	82(90.1%)	67(88.2%)	15(100.0%)	
Mild: |z| [1.64, 3)	5(5.5%)	5(6.6%)	0	
Missing	4(4.4%)	4(5.3%)	0	
**RVTLC_Z**				0.93
Normal: |z| <1.64	76(83.5%)	63(82.9%)	13(86.7%)	
Mild: |z| [1.64, 3)	11(12.1%)	9(11.8%)	2(13.3%)	
Missing	4(4.4%)	4(5.3%)	0	
**FEV1_Z**				0.25
Normal: |z| <1.64	70(76.9%)	56(73.7%)	14(93.3%)	
Mild: |z| [1.64, 3)	19(20.9%)	18(23.7%)	1(6.7%)	
Moderate:|z| [3,4)	2(2.2%)	2(2.6%)	0	
**FVC_Z_CAT**				0.67
Normal: |z| <1.64	72(79.1%)	59(77.6%)	13(86.7%)	
Mild: |z| [1.64, 3)	17(18.7%)	15(19.7%)	2(13.3%)	
Moderate:|z| [3,4)	2(2.2%)	2(2.6%)	0	
**FEV1/FVC_Z**				0.49
Normal: |z| <1.64	78(85.7%)	66(86.8%)	12(80.0%)	
Mild: |z| [1.64, 3)	13(14.3%)	10(13.2%)	3(20.0%)	
**FEF2575_Z**				0.09
Normal: |z| <1.64	74(81.3%)	64(84.2%)	10(66.7%)	
Mild: |z| [1.64, 3)	14(15.4%)	9(11.8%)	5(33.3%)	
Moderate:|z| [3,4)	3(3.3%)	3(3.9%)	0	

Supplementary Table 3. Baseline PFT Data with Z Scores.

DLCO = diffusing capacity for carbon dioxide (DLCO), TLC = total lung capacity, RV = residual volume, FEV1 = forced expiratory volume in one second, FVC = forced vital capacity, FRC = forced residual capacity, FEF25-75 = mid forced expiratory flow volumes between 25% and 75% of vital capacity

	All Patients	No ICU	ICU	P-value
	N=74	N=64	N=10	
**DLCO_Z**				**0.03**
Normal: |z| <1.64	50(67.6%)	46(71.9%)	4(40.0%)	
Mild: |z| [1.64, 3)	16(21.6%)	11(17.2%)	5(50.0%)	
Moderate:|z| [3,4)	2(2.7%)	1(1.6%)	1(10.0%)	
Missing	6(8.1%)	6(9.4%)	0	
**TLC_Z**				0.82
Normal: |z| <1.64	60(81.1%)	52(81.3%)	8(80.0%)	
Mild: |z| [1.64, 3)	6(8.1%)	5(7.8%)	1(10.0%)	
Missing	8(10.8%)	7(10.9%)	1(10.0%)	
**RV_Z**				0.48
Normal: |z| <1.64	63(85.1%)	54(84.4%)	9(90.0%)	
Mild: |z| [1.64, 3)	3(4.1%)	3(4.7%)	0	
Missing	8(10.8%)	7(10.9%)	1(10.0%)	
**RVTLC_Z**				0.27
Normal: |z| <1.64	59(79.7%)	50(78.1%)	9(90.0%)	
Mild: |z| [1.64, 3)	7(9.5%)	7(10.9%)	0	
Missing	8(10.8%)	7(10.9%)	1(10.0%)	
**FEV1_Z**				0.33
Normal: |z| <1.64	62(83.8%)	52(81.3%)	10(100.0%)	
Mild: |z| [1.64, 3)	11(14.9%)	11(17.2%)	0	
Moderate:|z| [3,4)	1(1.4%)	1(1.6%)	0	
**FVC_Z**				0.33
Normal: |z| <1.64	62(83.8%)	52(81.3%)	10(100.0%)	
Mild: |z| [1.64, 3)	11(14.9%)	11(17.2%)	0	
Moderate:|z| [3,4)	1(1.4%)	1(1.6%)	0	
**FEV1/FVC_Z**				0.27

Normal: |z| <1.64	67(90.5%)	57(89.1%)	10(100.0%)	
Mild: |z| [1.64, 3)	7(9.5%)	7(10.9%)	0	
**FEF2575_Z**				0.05
Normal: |z| <1.64	56(75.7%)	46(71.9%)	10(100.0%)	
Mild: |z| [1.64, 3)	18(24.3%)	18(28.1%)	0	

Supplementary Table 4. One-year Post-HCT PFT Data with Z Scores.

DLCO = diffusing capacity for carbon dioxide (DLCO), TLC = total lung capacity, RV = residual volume, FEV1 = forced expiratory volume in one second, FVC = forced vital capacity, FRC = forced residual capacity, FEF25-75 = mid forced expiratory flow volumes between 25% and 75% of vital capacity
